# Emerging approaches of wound healing in experimental models of high-grade oral mucositis induced by anticancer therapy

**DOI:** 10.18632/oncotarget.28091

**Published:** 2021-10-26

**Authors:** Ana Chor, Maria Adriana Skeff, Christina Takiya, Raquel Gonçalves, Marcos Dias, Marcos Farina, Leonardo R. Andrade, Valeria de Mello Coelho

**Affiliations:** ^1^Laboratory of Biomineralization, Institute of Biomedical Sciences, Health Sciences Center, Federal University of Rio de Janeiro, Rio de Janeiro 21941-902, Brazil; ^2^Laboratory of Immunopathology, Institute of Biophysics Carlos Chagas Filho, Health Sciences Center, Federal University of Rio de Janeiro, Rio de Janeiro 21941-902, Brazil; ^3^Laboratory of Catalysis for Polymerization, Recycling and Biodegradable Polymers (LCPRB), Institute of Macromolecules Professor Eloisa Mano (IMA), Technological Center, Federal University of Rio de Janeiro, Rio de Janeiro 21941-598, Brazil; ^4^Laboratory of Immunophysiology, Institute of Biomedical Sciences, Health Sciences Center, Federal University of Rio de Janeiro, Rio de Janeiro 21941-902, Brazil

**Keywords:** oral mucositis, inflammation, regeneration, polymers, tissue engineering

## Abstract

Clinical guidelines for oral mucositis (OM) still consist in palliative care. Herein, we summarize cellular and molecular mechanisms of OM ulceration in response to chemical therapies in animal models. We discuss evidenced anti-inflammatory and anti-oxidant drugs which have not been ever used for OM, such as synthetic peptides as well as cell therapy with mesenchymal stem cells; amniotic membranes, mucoadhesive polymers loaded with anti-inflammatory agents and natural or synthetic electrospun. These approaches have been promising to allow the production of drug-loaded membranes, scaffolds for cells encapsulation or guided tissue regeneration.

## INTRODUCTION

Mucositis refers to lesions caused along the gastrointestinal tract by antineoplastic agents while stomatitis includes a broad range of inflammatory conditions in the oral cavity only [1]. Despite differences between these terms, some researchers interchange their use when refers to OM, which is one of the most symptomatic and troublesome side effects of antineoplastic treatment [2]. The first clinical symptom of OM usually appears after one week of combined radio-chemotherapy as a reddish erythema of the oral mucosa, which evolves lately into ulcerations. In its severe form, OM results in deep and diffuse ulcerations causing pain and, possibly, loss of function [2].

Prolonged ulcerations along the cumulative doses of radio-chemotherapy causes: impairment in food intake; increased need for hospitalization, opioid use and feeding tube or parenteral nutrition; delays in treatment; risk factor for sepsis; and decreased quality of life along treatment [1, 3]. In addition to inflammation and formation of ulcerations in the oral mucosa, chemicals often cause side effects such as bone marrow suppression, renal, liver, cardiac muscle cells and intestinal toxicity. Upon injury, molecular mediators stimulate transcription factors that regulate the expression of pro-inflammatory cytokines that activate resident cells, local vascular cells and immune system cells in the site of injury [4–6]. Consequently, clinical symptoms including swelling, heat, redness, pain and loss of function may occur.

**Figure 1:**
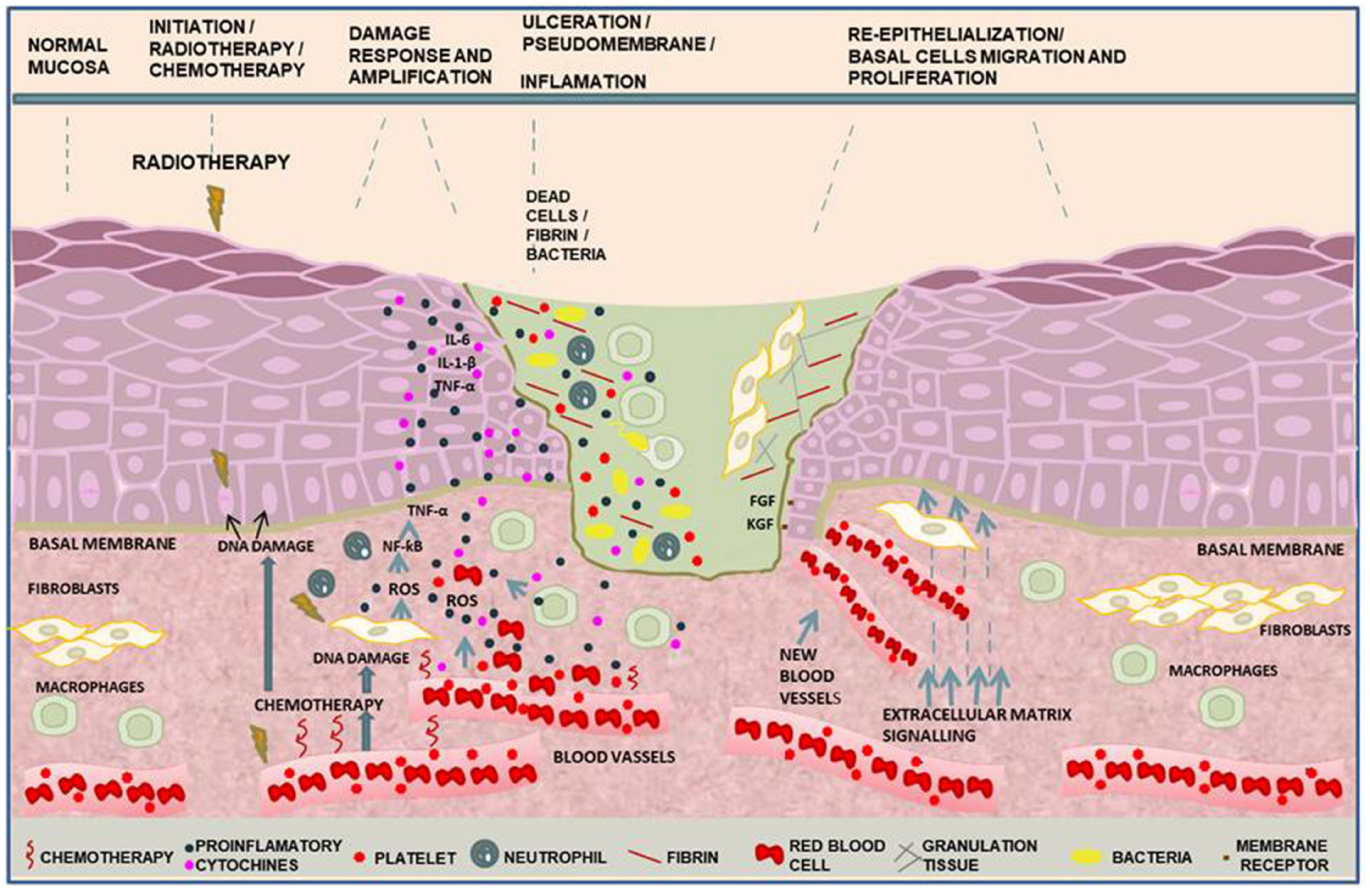
Molecular pathways in the phases of oral mucositis induced by cancer therapy. In the initiation phase, DNA damage in the basal epithelial cells and mesenchymal cells induced by anticancer treatment leads to reactive oxygen species (ROS) production and cell death. Injury response and amplification induced during radiotherapy and/or chemotherapy by necrotic molecules activate nuclear factor-κβ (NF-κβ) that regulates genes to IL-1-β, IL-6 and tumor necrosis factor α (TNF-α) pro-inflammatory cytokines that damage endothelial cells and fibroblasts. Endothelial membrane breakdown leads to extravasation of red blood cells and platelets to the site of injury resulting in clot formation. The recruitment of neutrophils and macrophages to the site of injury establishes the inflammatory process. Oral cavity bacteria and funghi colonize the clot containing dead cells onto the established ulceration. Bacteria cell wall products recruit macrophages towards the site of injury amplifying the damage. As soon as ROS production ceases, re-epithelialization occurs. Mesenchymal-epithelial cells interaction leads to growth factors release. Vascular endothelial growth factor (VEGF), platelet derived growth factor (PDGF), transforming growth factor (TGF-α), fibroblast growth factor (FGF) and keratinocyte growth factor (KGF), collectively induce new blood vessels formation, keratinocytes proliferation and epithelial cells differentiation.

OM has five distinct cytopathological phases considering the model developed by Sonis [2, 7] 1st, initiation; 2nd, damage response; 3rd, signaling and amplification; 4th, ulceration; and 5th, re-epithelialization. These phases were primarily based on morphological tissue modifications involving several biological mediators (Figure 1). In this regard, reactive oxygen species (ROS) contribute to OM initiation generating oxidative stress through a variety of biological processes, being the membrane lipid peroxidation, one of the most relevant [8]. As a response to ROS damage, antioxidant enzymes such as superoxide dismutase and catalase and antioxidant molecules, including ascorbic acid and glutathione, are produced locally [7–11]. Glutathione promotes free radicals scavenging, detoxification of xenobiotic and carcinogens, redox reactions and modulation of cytokines expression in injured tissues [12]. ROS induce changes in ceramide pathways related to the expression of specific matrix metalloproteinase (MMP) in submucosa cells [7, 11]. MMPs contribute to matrix degradation and interruption of signal transduction in epithelial progenitor cells causing epithelium layers breakdown [11–13].

In the second phase, TNF-α and IL-1β significantly increased in OM ulceration activates the protein complex nuclear factor kappa-light-chain-enhancer of activated B cells (NFκβ) and its translocation into the nucleus inducing gene expression of several pro-inflammatory cytokines [14].

Radio-chemotherapy in the oral mucosa decreases the expression of anti-apoptotic proteins β-cell lymphoma-2 (Bcl-2), myeloid cell leukemia 1 (Mcl-1), β-cell lymphoma-extra-large (Bcl-XL) and Bcl-w and A1, while increases the expression of the pro-apoptotic protein p53 [2, 7, 8, 15, 16]. These proteins regulate mitochondrial membrane permeability and caspase activation causing damages to DNA and proteins [17, 18]. In the third phase of the OM development, cell signaling promotes oxidative stress [7] (Figure 1). Fibroblasts and endothelial cells also release ROS to the extracellular milieu resulting in additional recruitment of neutrophils and monocytes to the site of injury amplifying the inflammatory response, which promotes additional epithelial injury and further ulcerative lesions [14, 15]. In the fourth phase, ulceration with pseudo-membrane formed by dead cell layers colonized by oral bacteria is a hallmark of severe OM caused by antineoplastic treatments that once invading small blood vessels lead to septicemia [19–21]. Bacteria cell wall products also attract monocytes/macrophages into the ulceration extending the inflammatory process [22]. In the last phase, re-epithelialization occurs after cancer treatment ceases. Growth factors, calcium and nitric oxide (NO) signaling as well as secretion of molecules by submucosal cells stimulate differentiation and proliferation of stem cell to regenerate the epithelial barrier. Migration of keratinocytes to the edges of the ulcer occurs [11, 23, 24].

**Figure 2:**
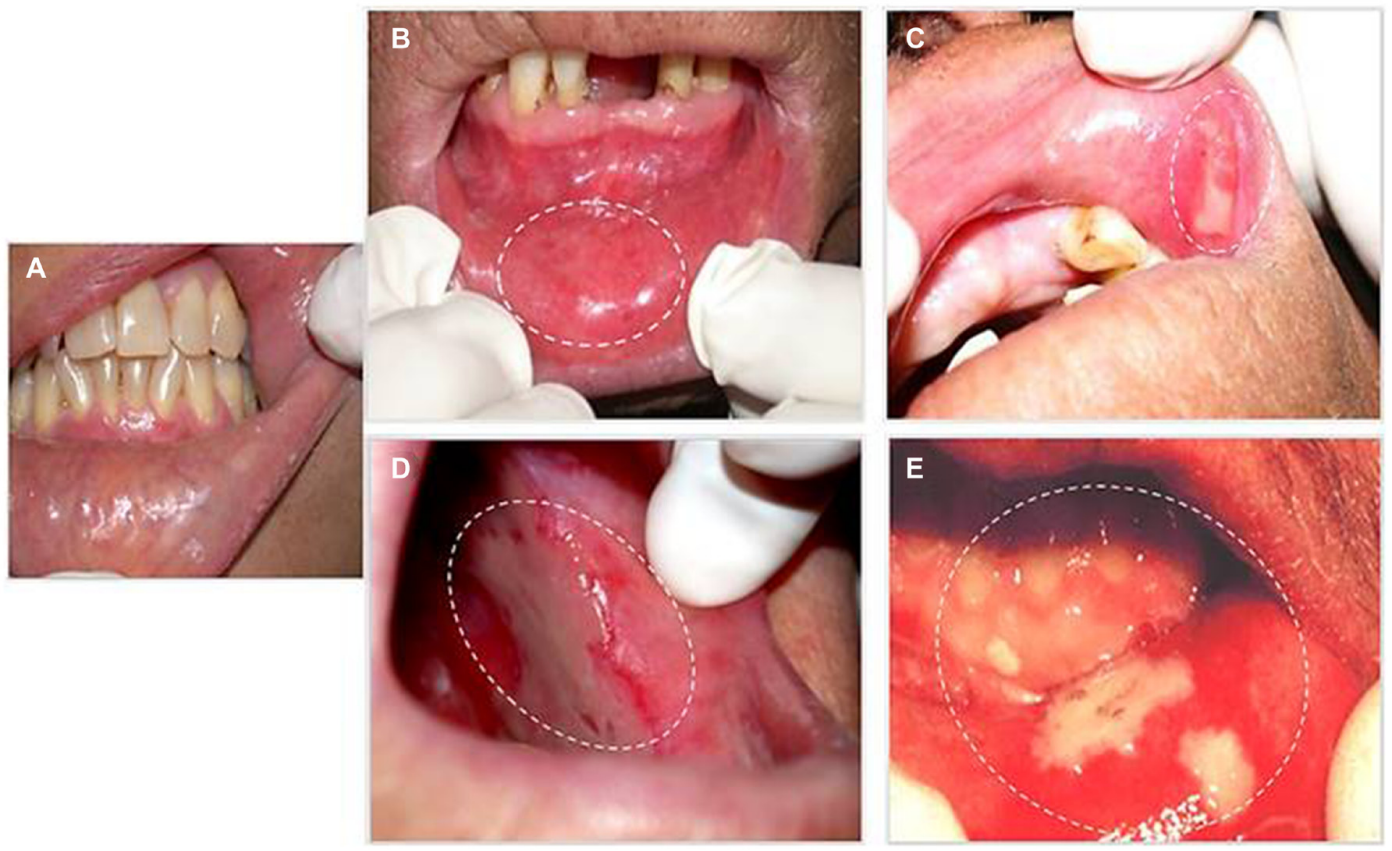
Evolution of human OM during chemoradiotherapy. (**A**) Initial aspect of lower labial mucosa without any change; (**B**) Mucosa erythema; (**C**) Initial break in the mucosa represented by a discrete ulceration covered by a pseudomembrane; (**D**) Confluent ulcerations covered by a fibrinous pseudomembrane; and (**E**) Areas of necrosis. Informed consent was obtained from the patients to publish images related to diagnosis of oral mucositis.

The scores of clinical evolution of OM are established by the World Health Organization (WHO) and the Common Terminology Criteria for Adverse Events (CTCAE), which present the most used criteria to describe the toxicity in humans [25]. These scores are related to the aforementioned molecular pathways described in animal models and are clinically illustrated in Figure 2. The WHO scale determines the objective, subjective and functional aspects related to the scores (0 to 4) in one scale. On the other hand, CTCAE version 3 punctuates the clinical findings and the symptoms in two distinct scales, both highlighting the grade 4 with life-threatening consequences and tissue necrosis [25].

Considering the most symptomatic phase of OM as a life-threating condition [4, 6], researchers are looking forward for innovative approaches. Here, we focus on the emerging experimental therapies showing cellular and molecular evidences in animal models that have not ever been used to treat OM ulcerations. We discuss data regarding the use of anti-inflammatory and anti-oxidant drugs, synthetic peptides and the potential use of therapies with mesenchymal stem cells, amniotic membranes, natural or synthetic electrospun polymers loaded with anti-inflammatory agents (Table 1) for OM healing that might contribute for future human clinical trials in the area.

**Table 1:** Emerging approaches for OM treatment in experimental models

Treatments	Experimental models	Main effects	Ref.
S-nitrosoglutathione (GSNO)	5-FU induced OM in hamster	Reduced expression of iNOS in the site of OM	[42]
Azilsartan (AZT)	5-FU induced OM in hamster	Reduced TNF-α and IL-1β Levels. Increased IL-10 Levels and Upregulated expression of VEGF, FGF, KGF, and TGF-α	[45]
Telmisartan (TELM)	5-FU induced OM in hamster	Reduced expression of COX2, MMP-9 and iNOS in the site of lesions Reduced gene expression of NF-κβ p65, TNF-α, IL-1β and TGF-β / Smad 2/3 signalling.	[53]
Olmesartan (OLME)	5-FU induced OM in hamster	Reduced MIF along with MPO activity, malondialdehyde (MDA), iNOS and ERK1/2. Reduced gene expression of IL-1β, TNF-α, NFκβp65, MKP1 and ACE2. Increased expression of IL-10, FGF-2 and TGF-β in the site of lesion	[54]
Rosiglitazone (RGZ)	5-FU induced OM in mouse	Inhibited activation of NF-κβ and decreased expression of TGF-β, IL-1β and MMP-2 and p53 apoptotic marker. Increased expression of collagen.	[55]
Rosiglitazone (RGZ)	Radiation-induced OM in mouse	Increased cellular proliferation and regeneration of the epithelial barrier Prevented Oxidative stress induced by ROS. Increased TGF-β expression associated with anti-oxidant and anti-inflammatory actions	[56]
Salvia miltiorrhiza Bunge (SM)	5-FU induced OM in hamster	Promoted antioxidant effects against scavenging 2-diphenyl-1-picrylhydrazyl (DPPH) free radicals *in vitro*. Suppressed ROS production in CCL-138 cell line. Decreased number of apoptotic cells in the lesions. Reduced expression of NF-κβ and cleaved caspase-3 *in vitro* and *in vivo*. Decreased expression of IL-1β and TNF-α proinflammatory cytokines and decreased NF-κβ activation.	[81]
Hesperidin (HSP)	5-FU induced oral stomatitis in hamster	Inhibited expression of TNF-α, COX2, inducible-NO-synthase and prostaglandin E2	[82]
Apigenin	5-FU induced OM in hamster	After 5 days, animals presented intense polymorphonuclear inflammatory infiltrate. After 10 days, treatment resulted in greater re-epithelialization and diminished inflammatory cell counting in the site of injury. Apigenin exerted an antioxidant effect in OM compared to classic treatment with dexamethasone.	[83]
1-Palmitoy-2-linoleoyl-3-acetyl-rac-glycerol (PLAG)	5-FU induced OM in hamster and mouse	Increased number of circulating neutrophils and decreased expression of IL-6, TNF, and IL-1β inflammatory cytokines. Reduced extravasation of neutrophils. Decreased ulceration, fibrosis, and festering wounds in PLAG-treated hamsters ameliorating the inflammatory process.	[84]
Human Gingiva-derived Mesenchymal Stem Cells	5-FU induced OM in mouse	Mesenchymal-epithelial transition and faster re-epithelialization in tongue ulcerations.	[86]
Human bone marrow derived mesenchymal stem cells (hMSCs)	Radiation/chemical-induced OM in mouse	hMSCs transfected with CXCR2 induced down regulation of TNF-α, IL-1β and IL-6 pro-inflammatory cytokines and decreased recruitment of pro-inflammatory cells into the site of lesion	[87]
Adipose derived mesenchymal stem cells (aMSCs)	Radiation-induced OM in mouse	5 doses of 2.5 million of cultured syngeneic aMSCs reduced 72% of OM duration Decreased expression of TNF-α, IFN-γ and IL1-β. Increased expression of IL-10.	[90]
ElectrospunKet-loaded Eldragit	acetic acid induced oral mucosa lesions in rabbits	Reduced clinical severity of simulated mucositis. Suppressed inflammatory response and stimulated healing process in rabbits.	[106]
Amniotic membrane (AM)	5-FU-induced OM in rats	AM was biocompatible and stimulated tissue repair in the 5-FU-induced OM. Increased cellular proliferation and neovascularization.	[113]
Chitosan-alginate film containing royal jelly (RJ)	5-FU induced OM in hamster	MPO expression decreased in RJ-loaded chitosan-alginate film treated ulcerations; TNF-α and IL-1β proinflammatory cytokines decreased after 8 days of RJ treatment. RJ treatment exerted anti-inflammatory and antioxidant action when associated to a polymer dressing material.	[115]
Electrospun hGH-loaded Eudragit coated with chitosan	acetic acid induced oral mucosa lesions in beagles	enhanced cellular proliferation and larger amounts of hGH released.	[117]

### Current approaches used to heal OM

Life expectancy is rising and the older population is growing. In the next years, cancers related to aging are expected to grow and the side effects of OM as well. A high percentage of patients with head and neck tumors suffer from adverse effects of OM. Currently, several interventions are used for OM treatment however there is no gold standard. Therapies currently used in humans that ameliorate OM include low-level laser therapy (LLLT) cryotherapy and palifermin, which is recombinant keratinocyte growth factor, KGF. Other interventions also with uncertain results included the use of with aloe vera, amifostine, glutamine, G-CSF, honey, laser, polymyxin/tobramycin/amphotericin (PTA) paste or tablets, and sucralfate [26].

Although animal models have limitations, their use has been a relevant source of knowledge to understand critical morphophysiological processes when sampling is not possible in humans and to allow translation from bench to clinical trials [27]. In this regard, recent clinical practice guidelines for OM recommend therapies [28] that have been previously tested in experimental models such as low-level laser therapy (LLLT), amifostine, glutamine and various herb based agents [16, 29, 30].

In particular, LLLT, which is a type of photobiomodulation, promotes pain relief due to specific anti-inflammatory effects and accelerates wound healing contributing to manage deleterious effects of oral mucositis in cancer patients [31]. This phototherapy that was previously verified to be efficient in clinical trials for OM management has been applied as a preventive therapy due to its potential biological mechanism of action. It stimulates endogenous chromophores resulting in both photochemical and photophysical reactions when applying wavelengths between 600 and 1100 nm [32–34]. In fact, red or near infrared (NIR) light penetrates through cell membrane targeting mitochondria promoting light absorption by the enzymatic chromophore cytochrome c that induces the electron transport chain during ATP production. Consequently, ATP increase induces gene transcription and cell proliferation [33, 34]. LLLT has been indicated as preventive therapy for head and neck cancer patients and for receptors of bone marrow transplantation by guidelines of the mucositis study group of the Multinational Association of Supportive Care in Cancer and International Society of Oral Oncology (MASCC/ISOO) and by the MASCC/ISOO Clinical Practice Guidelines for the Management of Mucositis Secondary to Cancer Therapy [28, 29]. However, given the lack of protocols to treat OM ulcerative lesions using LLLT [35] or other treatment modalities [28, 29], experimental studies are still crucial to test new approaches to this very symptomatic condition, such as the studies discussed in the present review.

### Animal models for OM

OM is mainly developed due to head and neck cancer treatment with radiochemotherapies and mouse and hamsters have been the most used animal models to study its healing [3]. These models allowed the knowledge of the pathways of OM induced by anticancer treatments [3, 21, 27], in particular the understanding of epithelial repopulation, and the acceleration process of healing after drug applications, promoting ameliorations to head and neck and hematological cancer patients [36].

Although the molecular and cellular complexity of cancer is different between human and mouse, there are genomic and physiological similarities between them which support its use as models in biological research and pre-clinical studies [37]. In this context, the use of mice in pre-clinical studies of cancer research has been largely explored to characterize biological processes following radiotherapy and chemotherapy besides the effects of different drugs in the organism. However, differently, in the model of OM there is no malignant lesion in the oral cavity of animals, being only studied the development and repair of oral lesions induced by radiochemoterapy used in cancer treatment.

Syrian hamsters and other rodents have been used to develop OM in response to radiation only, radiochemotherapy or to chemotherapy using 5-Fluorouracil (5-FU), which is incorporated into the RNA and DNA using the same chemical route as uracil, causing harmful effects on cellular metabolism and viability [3, 8–10, 38]. The hamster, owing to its vascularized cheek pouch is considered an excellent model to reproduce the lesion severity in the oral mucosa as occurs in humans with OM. In this regard, Sonis was the first to use this model to test therapies to accelerate OM healing in head and neck or hematological cancer patients [23]. Currently, other research groups have also used hamster animal model to evaluate development and pathways of OM following radiation, chemotherapy or chemoradiation [39, 40].

In this review, we selected studies published in the last years at PubMed and Web of Science database using the terms “oral mucositis” and “stomatitis”. Our exclusion criteria were: a) Research articles using experimental models without morphological analyses; b) Clinical trials articles, since they do not show morphological analyses; c) Experimental research articles that tested anti-inflammatory drugs, antioxidants, polymers and other therapies that have already been tested in clinical trials and have been recommended in clinical practice guidelines for OM.

### Emerging approaches to heal OM in animal models

#### Mucoadhesive gel loaded with S-nitrosogluotathione (GNSO)

Mucoadhesive gel loaded with S-nitrosogluotathione (GSNO) antioxidant, the S-nitrosated derivative of glutathione (GSH), has a crucial role in the biological effects of NO [41]. GSNO stimulates blood flow increase and modulates the immune response during wound healing [41, 42]. Skeff and collaborators (2014) worked with a 5-FU-induced OM in hamster model to test the effects of GSNO on ulceration healing. Different concentrations of GSNO (0.5, 1.0 and 2.0 mM) were mixed with hydroxypropylmethylcellulose (HPMC) resulting in an adhesive gel applied twice a day up to 14 days onto ulcerations 1 h before 5-FU administration [42]. Interestingly, 2.0 mM of GSNO induced a delayed OM regeneration [42]. The authors also showed that 0.5 mM and 2.0 mM HPMC/GSNO reduced the expression of inducible nitric oxide synthase (iNOS) in the site of OM ulcerations suggesting a protective effect of GSNO against iNOS. It is known that pro-inflammatory cytokines induce NO and free radicals dependent of iNOS activity resulting in the activation of innate immune response [42]. Possibly, NO and reactive oxygen products reaction led to peroxynitrite formation [41]. The authors speculated that the therapeutic concentrations of GSNO should remain in the micromolar range, probably below 500 μM [41]. Exceeding this concentration, iNOS-dependent cytotoxic NO would be expected although the cytotoxicity would vary among distinct types of tissues [43]. It is also important to highlight that hamsters have an oral microflora similar to humans [8]. Experimental research in hamster model revealed a 300-fold increase in bacteria in the site of OM ulcerations, in comparison with the normal epithelium [3, 8, 42]. Skeff and collaborators (2014) showed an increase in the number of Gram-negative bacteria in the group of 5-FU-induced OM [42]. However, periodontal pathogens including P. gingivalis and T. forsythia were reduced after treatment with 0.5 mM HPMC/GSNO. These results indicate the potential of HPMC/GSNO treatment for OM, since P. gingivalis is considered one of the main pathogens in the oral cavity leading to delayed wound healing [42].

#### Angiotensin receptors blockers (ARBs)

ARBs known as Ang II receptor type 1 (AT1R) antagonists have been studied in the field of dentistry due to its anti-inflammatory effects [43]. Ang II, involved in the regulation of the cardiovascular system homeostasis, stimulates ATR1 resulting in upregulation of pro-inflammatory cytokines, neutrophil infiltration, reactive oxygen species production and NF-κβ activation [44]. In experimental periodontitis, a chronic infectious and inflammatory disease, and gastrointestinal mucositis, ARBs suppress TNF-α-induced activation of the NF-κβ classical pathway and gene expression of NF-κβ p65 subunit, which induces gene transcription of pro-inflammatory mediators in vascular endothelial cells [43, 45, 46]. In other diseases, ARBs show similar signaling pathway of 5-FU-induced pro-inflammatory cytokines [47–50]. In experimental models, ARBs suppress pro-inflammatory cytokines *in vitro* and *in vivo* [50, 51]. Indeed, ARBs in experimental models of OM decrease inflammation and oxidative stress while increase the levels of IL-10 anti-inflammatory cytokine as well as of growth factors associated with granulation tissue formation and wound healing [45].

Azilsartan medoxomil (AZT) is another ARB approved by the US Food and Drug Administration for oral treatment of hypertension that has been tested in periodontitis as an alternative treatment to control inflammatory symptoms in adults and elderly people [43]. In Wistar rats with periodontitis, AZT at 5 mg/kg daily for 10 days reduced neutrophil counting in the injury sites [43]. When using AZT at 1, 5 or 10 mg/kg orally 30 min before the 5-FU and then daily for 10 days in hamster model for OM [45], 1 mg/kg was the only one that showed efficacy. While the control with saline scored 5, AZT 1 mg/kg reduced significantly the number of neutrophils as well as local TNF-α, IL-1β and IL-10 expression, showing score of 2; and increased the expression of VEGF, FGF, KGF and TGF-α, all involved in the healing process [43–45]. AZT proved to be biologically safe in combination with 5-FU-induced OM in hamsters and promising to be used with 5-FU-induced OM in primates before clinical trials in humans [45].

Telmisartan (TELM) is an ARB that inhibits Ang II action and presents a pleiotropic anti-inflammatory effect in regulating blood pressure and atherosclerotic lesions in patients [52]. Barbosa and collaborators (2018) tested the regulatory effect of TELM on the expression of NFκβ-dependent inflammatory genes, using 5-FU-induced OM hamster models [53]. Histopathological and macroscopic analyses revealed that TELM administered orally at 5 and 10 mg/kg prevented OM development induced by 5-FU in hamsters, showing reduced infiltration of inflammatory cells and hemorrhagic areas, vasodilation and absence of abscesses and ulcers, indicating score of 2. TELM at 1 mg/kg had a score of 3 while the 5-FU-induced OM group that did not receive any treatment with TELM showed a score of 4 [53]. TELM at 10 mg/kg reduced the expression of cyclooxygenase 2 (COX2), MMP-9 and iNOS in the oral mucosa of hamsters previously treated with 5-FU, in comparison with the control with saline alone [53]. TELM at 5 or 10 mg/kg reduced gene expression of NF-κβ p65, TNF-α, IL-1β and TGF-β/Smad2/3, highlighting its beneficial effects for experimental OM. At 10 mg/kg, TELM increased mRNA expression of peroxisome proliferator activation factor γ (PPAR γ) and reduced mRNA expression of NFκβ p65 and STAT 1 induced by 5-FU in the oral mucosa, as well as the immunoreactivity for NFκβ p65 and iNOS in the site of lesions [53]. These results indicate that TELM might exert a protective effect on OM induced by 5-FU treatment [53].

Olmesartan (OLME) is an ATR1 blocker that was shown to present antioxidant and anti-inflammatory actions in different experimental models [46]. OLME was previously tested in a methotrexate-induced intestinal mucositis model, demonstrating promising results [46]. OLME was administered by oral gavage at 1, 5 or 10 mg/kg for 10 days in hamster model of 5-FU-induced OM. At 1 or 5 mg/kg OLME treatment showed erythema, hyperemia, hemorrhagic areas and extensive ulceration with signs of cellular inflammation as well as a prevalence of neutrophils, edema, ulcers and abscesses. However, OLME at 10 mg/kg resulted in less severe lesions, with discrete erythema but no ulcers and faster re-epithelialization of OM ulcerations in comparison with lower concentrations used [54]. The authors hypothesized that OLME also prevented lipid peroxidation, increased glutathione (GSH) levels and superoxide dismutase (SOD) enzyme activities in the injury sites. At 10 mg/kg, OLME possibly might downregulate the extracellular signal-regulated kinase (ERK1/2), which is activated by cellular oxidative stress besides reduce IL1β expression [54]. Moreover, OLME might downregulate mitogen-activated protein kinase (MKP1), which binds to ERK1/2 regulating cytokine gene expression [46]. OLME reduced myeloperoxidase (MPO) activity, malondialdehyde (MDA), iNOS and ERK1/2 as well as the expression of TNF-α, IL-1β, NFκβp65, MKP1 and angiotensin converting enzyme 2 (ACE2) as well the expression of macrophage migration inhibitory factor (MIF), which is associated with macrophages switch from M1 proinflammatory to M2 proliferative phenotype during wound healing. The authors verified that OLME at 10 mg/kg promoted the formation of granulation tissue in the injury site of 5-FU treated hamsters, which is consistent with the initial steps of wound healing process [54]. The expression of IL-10, FGF-2 and TGF-β in the site of lesion following OLME treatment at 10 mg/kg reinforced the hypothesis of macrophage switch from M1 to M2 at the site of OM [54]. Altogether, OLME treatment at 10 mg/kg decreased the oxidative stress and inflammation and stimulated fibroblasts activity and collagen deposition, recovering 5-FU-induced OM of hamsters.

### Rosiglitazone (RGZ)

RGZ is an intracellular ligand for PPAR-γ transcription factor that exerts antioxidant and anti-inflammatory activities. Currently, RGZ is used to control hyperglycemia in type II diabetes mellitus [55, 56]. Additionally, it has been reported that the thiazolidinedione family of PPARγ agonists, including troglitazone (TGZ), pioglitazone (PGZ) and RGZ, have antitumor effects [57, 58]. Recently, Fujita (2017) showed the antitumor effect of TGZ in pancreatic cancer cells *in vitro* and *in vivo*, suggesting that TGZ treatment is a potential approach for pancreatic cancer [58].

One of the ways that PPARs regulate inflammation is through a transrepression mechanism, where other transcription-factor, such as NF-κβ, is repressed to activate the expression of pro-inflammatory cytokines [55]. Consistent with the RGZ effects, Sottili and collaborators (2017) tested RGZ in a 5-FU-induced OM in mouse models. RGZ was administered at 5 mg/kg/day by oral gavage, starting 24 hours before the 5-FU induction for 15 days [55]. The authors demonstrated that RGZ inhibited the activation of NF-κβ protein leading to a reduced expression of TGF-β and IL-1β cytokines in the site of lesions. In addition, RGZ significantly inhibited the expression of MMP-2 and restored the expression of collagen [55]. Regarding genetic modifications, RGZ decreased the expression of the apoptotic marker p53 in 5-FU-induced OM group. These results indicated that RGZ might be a potent drug to modulate the molecular pathways of 5-FU-induced OM, thus suggesting its future application in oncologic patients [55].

Mangoni et al. [56] also investigated the effects of RGZ in the prevention of radiation-induced OM but in murine models. Radiation was applied at a single dosage of 16.5 Gy at the snout of animals. RGZ treatment at 5 mg/kg/day was administered by oral gavage, starting 24 h before irradiation up to 12 or 23 days. Results showed an effective antioxidant effect on radiation-induced OM. Considering the macroscopic analyses, lower OM scores were recorded for RGZ treatment groups in comparison with the control group of animals. Morphological analyses showed reduced inflammatory cells infiltration and edema formation in the site of injury. In addition, RGZ stimulated cellular proliferation contributing for the regeneration of the epithelial barrier. Oxidative stress induced by ROS production was prevented by the increase of catalase in the group treated with RGZ. Moreover, RGZ-treated animals re-established the tissue expression of TGF-β in the site lesions. The authors concluded that RGZ exerted a protective effect at tissue level due to its anti-oxidant and anti-inflammatory action during the course of radiation-induced OM [56].

### Medicinal herb derivatives

WHO and worldwide regulatory agencies, including FDA, the Canada Vigilance Program, and ANVISA, are working towards setting up current standards and regulations for herbal medicinal products [59–63]. To have new efficacious NHP approved, preclinical studies using plant-derived extracts in animal models, including tests for OM induced by radio-chemotherapy are being performed [64–74].

Natural health products (NHP) have also been studied mainly because of their antioxidant, anti-inflammatory and healing effects as well as anticancer activity [64, 75–78]. In this regard, Buccal Bullfrog (Rana catesbeiana Shaw) oil emulsion was successfully used as a mucoadhesive system to treat oral candidiasis [79]. In addition, curcumin diferuloylmethane, a polyphenolic compound isolated from the rhizomes of the dietary spice turmeric (Curcuma longa), has been studied in experimental models and clinical research particularly for cardiometabolic health and dementia due to its anti-oxidant and anti-inflammatory properties [80].

Salvia miltiorrhiza (SM) has been used in Korea, China and Japan for the treatment of various diseases, including: coronary heart disease; cerebrovascular disease; Alzheimer’s and Parkinson’s diseases; chronic renal deficiency; hepatocirrhosis; cancer and bone loss [81]. Kim et al. treated human pharyngeal cell lines (Detroit 562, ATCC CCL-138) with different concentrations of SM (1, 5, 10, 50 or 100 μg/mL) alone or in combination with 10 μM of 5-FU *in vitro* [81]. SM promoted antioxidant effects against scavenging 2-diphenyl-1-picrylhydrazyl (DPPH) free radicals and suppressed ROS production by CCL-138 cell line suggesting a protective effect on mucosal injury [81]. The treatment with different SM concentrations (100, 500 or 1,000 mg/kg) in hamsters previously treated with 5-FU for 14 days revealed a decrease in apoptotic cells in the lesions of SM-treated groups, as verified by terminal deoxynucleotidyl transferase (TdT) dUTP Nick-End Labeling (TUNEL) assay. SM also reduced NF-κβ expression and cleaved caspase-3 both *in vitro* and locally *in vivo* [81]. After 14 days of SM treatment, local expression of IL-1β and TNF-α as well as of NF-κβ decreased in the 5-FU-induced OM in hamsters. In addition, SM increased granulation tissue formation and cell growth *in vivo* [81]. These data indicate that SM might exert a protective effect against 5-FU-induced OM cytotoxicity.

Flavonoids help absorption and functional activity of vitamin C avoiding its oxidation and accelerated degradation [65]. Hesperidin (HSP), a flavonoid found in citrus fruits is an antioxidant molecule already tested in cancer models, Alzheimer, atherosclerosis diseases and others [82]. Yoshino and collaborators (2016) analyzed the activity of α-glucosyl hesperidin (HSP-G) in 5-FU-induced oral stomatitis in hamsters [82]. HSP-G treatment applied up to 21 days starting 5 days before 5-FU-induction showed a decrease in the local expression of TNF-α, COX2, iNOS and prostaglandin E2 [82]. In parallel, human HSC-3 oral squamous carcinoma cell line treated with 5-FU before the addition of HSP-G at different concentrations (0.01, 0.1 or 1.0 mg) showed ROS scavenger reaction without interfering with the antitumor effect of 5-FU *in vitro* [82].

The bioflavonoid Apigenin also may exert chemo-preventive effects due to its anti-inflammatory and antioxidant potential action and to reduce the expression of IL-1, IL-6, IL-8 and TNF-α pro-inflammatory cytokines [83]. Studies reported that flavonols and flavones may act by suppressing COX-2, which is involved in the production of prostaglandins and thromboxanes at the beginning of the inflammatory process in the site of injury [83].

Palmitoyl-2-linoleoyl-3-acetyl-rac-glycerol (PLAG) is a lipid isolated from the antlers of Sika deer (Cervus nippon Temminck) currently used in oriental medicine and synthesized as monoacetyl-diglyceride [84, 85]. Lee and collaborators (2016) reported that PLAG at 50 mg/kg/day regulated neutrophil transmigration in mice model [85]. This same group tested PLAG to prevent OM inflammation induced by anticancer treatment in hamster and mouse models [85]. Oral administration of PLAG at 250 mg/kg/day, after hamsters received scratching in the tongue or treatment with 5-FU, increased the number of circulating neutrophils and decreased IL-6, TNF and IL-1β expression in lesions. In addition, it reduced neutrophils extravasation and decreased volume and weight of cheek pouch ulcers in these animals [85]. Severe ulceration, fibrosis, and festering wounds were observed in the hamsters that received scratching or were treated with 5-FU in comparison with the group treated with PLAG, which ameliorate of the inflammatory process [85]. By day 13, 5-FU and scratching hamsters groups exhibited a 15% decline in body weight, which is characteristic of cachexia, a complication associated with both antineoplastic treatment and mucositis. PLAG-treated hamsters from the 5-FU-induced and scratching groups showed only 5% of decrease in body weight, indicating cachexia restoration [85]. The anti-inflammatory effect of PLAG associated with its role inducing regeneration in the site of injuries decreased OM scores indicate it may be a useful adjuvant for antineoplastic treatment.

### Cell therapy

Cell therapy using mesenchymal stem cells (MSCs) is a promising approach in the field of regenerative medicine [86]. MSCs can be isolated from bone marrow or sources, including from oral cavity tissues [87, 88]. Zhang and collaborators used gingival-derived mesenchymal stem cells (GMSCs) to treat 5-FU- induced OM in experimental Balb/c mice [88]. On the fourth day after 5-FU-induced OM, animals were treated with intravenous infusion of fluorescent-labeled GMSCs. Histological analyses in untreated mice evidenced epithelial surface atrophy, epithelium disruption and basal membrane loss in the tongue, which is characteristic of ulcerations while treated mice presented numerous labeled GMSCs and epithelial barrier recovery in their tongue. Labeled GMSCs in the epithelium suggested mesenchymal-epithelial transition and faster re-epithelialization of the tongue [88]. Shen and collaborators also produced OM lesions in tongue of mice following 16 Gy radiation exposures (a dose of 1.6 Gy/min) through a 10-mm-diameter hole [89]. Lesions were treated with bone marrow derived MSCs. Oral ulcers were chemically induced by placing a 3 × 3-mm round filter paper soaked with 70% acetic acid on the buccal mucosa for 30 s. The ulcerations were treated using a construct MSCsCXCR2 obtained after MSCs were infected with a lentiviral vector encoding the CXCR2 chemokine receptor, which binds to CXCL2 that is upregulated in oral tissue submitted to radiation and chemotherapy. The presence of MSCsCXCR2 in the injuries decreased production of ROS and mRNA expression of TNF-α, IL-1β and IL-6 as well as decreased recruitment of pro-inflammatory cells [89]. MSCsCXCR2 accelerated wound healing due to anti-oxidant and anti-inflammatory effects in the damaged area of OM [89]. Moreover, Maria and collaborators (2016) evaluated in a radiation-induced OM mouse model the effect of adipose-derived mesenchymal stem cells (aMSC), which are multipotent progenitor cells of the adipose tissue stromal vascular fraction with anti-inflammatory/immunomodulatory activities [90]. Experimental radiotherapy-induced OM in mice treated with aMSCs by intraperitoneal injections (5 doses of 2.5 million of cultured syngeneic aMSCs) resulted in 72% reduction in OM duration associated with a decreased expression of TNF-α, interferon-γ and IL1-β and an increased expression of IL-10 in the oral mucosa. Weight gain, another improvement in mice with irradiation-induced OM, was followed by hydration and nutritional status recoverage [90].

### Tissue engineering approach

The use of natural or synthetic scaffolds free or with the addition of cells, biomolecules or nanoparticles have been applied for tissue regeneration [91–112]. For this end, advances in the application of biomaterials including natural organic polymers, such as alginate and chitosan or inorganic polymers, as synthetic biomaterials, hydroxyapatite or polyesters, among others, are under investigation [91–99]. In the last years, biomaterials have been produced using the electrospinning technology to arrange fibers in a 3D fashion to mimic the extracellular matrix scaffold [98, 99, 103–105]. The electrospinning process is considered an innovative and versatile technique to produce biocompatible polymers in the shape of membranes, foams or tubes [99]. FDA approved scaffolds proven to be biocompatible. In addition, their biodegradability and nontoxic properties give support to their use as dressing materials to improve the healing of ulcerative lesions. Synthetic biomaterials may have great regenerative potential because they can be loaded with active molecules and/or cells [103–107]. The addition of progenitor cells or molecules onto these scaffolds is extremely promising for the field of tissue engineering and regeneration [102, 109–111].

**Figure 3:**
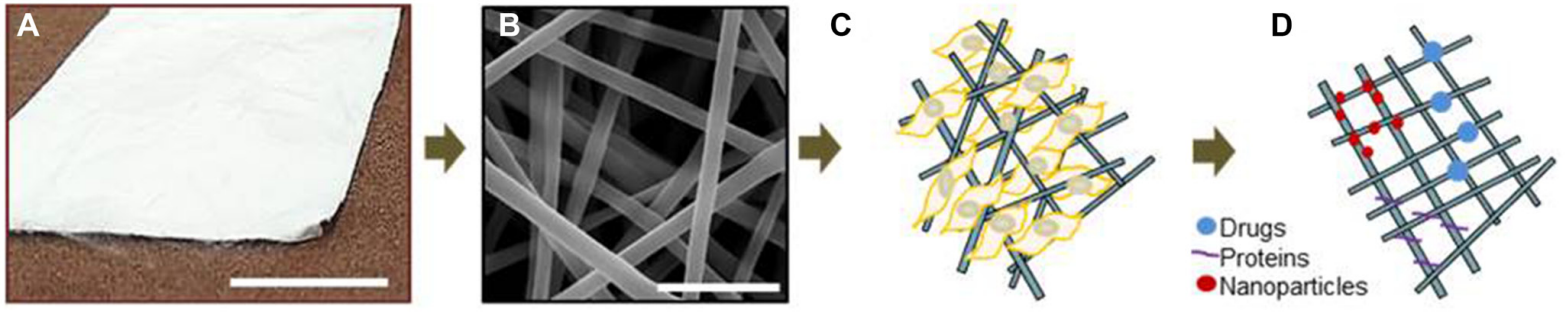
Electrospun PLGA scaffold for tissue engineering application. (**A**) Randomly electrospun microfibers in the format of membrane or film, created by Dr. Raquel Pires Gonçalves and Dr. Marcos Dias Lopes at the Institute of Macromolecules Professor Eloisa Mano of the Federal University of Rio de Janeiro. Scale bar: 4.5 cm; (**B**) Scanning electron microscopy image of PLGA membrane showing randomly oriented microfibers. Scale bar: 2.5 μm; (**C**–**D**) Schematic representation of the polymeric membranes with adhered cells, drugs or proteins to be carried out to specific biological sites. Scale bar: 2.5 μm. Dr. Marcos Farina obtained the images using the scanning electron microscope (FEI Quanta 250 at 15KV, 2048 x 2048 pixels) at Federal University of Rio de Janeiro.

An electrospun membrane, which may function as a synthetic scaffold biomaterial for dressing is shown in Figure 3. The membrane may be loaded with drugs, proteins, nanoparticles or cells [99, 101, 105–108, 111, 112]. Drug-loaded materials exert better results to be applied locally, since the drug is delivered in higher amounts to the target tissue due to sustained drug release, improving its local effect and decreasing systemic absorption [106, 108]. Among various topical non-steroidal anti-inflammatory drugs (NSAIDs), Ketoprofen (KET) has nontoxic property and exhibits stable interaction onto the oral mucosa lesions for regeneration [106]. Moreover, it blocked the synthesis of human PGE2 *in vitro* [106]. KET also inhibited monocytes and macrophages, cells that produce PGE2 [106].

Focusing on translation for treatment of cancer patients with OM, Reda and collaborators used electrospun KET-loaded Eudragit to accelerate the healing process of acetic acid induced oral mucosa ulcerations in rabbit models [106]. Electrospun KET-loaded Eudragit possess mucoadhesive properties due to many COOH groups, which contributes for hydrogen bond with the mucosal tissue [106]. This polymer potentially interacts with the mucosa after fluid uptake through the presence of numerous nanometer-sized interfibrilar pores causing mucoadhesion [106]. Eudragit composition of 20% (w/v) EL-NF loaded with 20% (w/v) KET reduced the clinical severity of OM since it suppressed the inflammatory response and induced the regenerative process of rabbits’ ulcerations [106]. Choi and collaborators (2015) showed electrospun Eudragit-loaded human growth hormone (hGH) membranes coated with chitosan produced for experimental ulcer treatment, suggesting future application in cancer patients [113]. In this study, electrospun anionic Eudragit L, composed of methacrylic acid and methyl methacrylate (1:1) was further coated with chitosan to control the dissolution rate of the electrospun carrier and the release rate of the drug. These membranes were used as dressing materials for oral ulcerations induced by acetic acid in beagle dog models. After 3 days of ulcer dressing, an identical second ulcer dressing was performed for 4 days and then mucous membranes were cut out after 7 days for histological analysis. Morphological analysis revealed enhanced cell proliferation due to the larger amount of hGH released from membranes-loaded with 0.5% of hGH, in comparison with the amount of 1.0% hGH. The results evidenced a larger amount of hGH released from membranes with lower concentration of the hormone [113]. More recently, our group produced Poly (Lactic-Co-Glycolic Acid)-PLGA electrospun scaffolds for oral mucosa regeneration aiming future application as dressing material for oral ulcerative lesions in head and neck cancer patients [114].

### Amniotic membrane (AM)

AM is a natural scaffold used for growth, migration and adhesion of epithelial cells to accelerate the healing process of lesions due to its biocompatibility [115]. AM corresponds to the innermost layer of placenta. It is composed of a single epithelial layer, a basement membrane and an avascular stroma with growth factors, cytokines and some bioactive substances [115, 116]. These factors contribute to its anti-inflammatory, anti-fibrotic, anti-microbial, anti-scarring and low immunogenicity properties as well as to promote neovascularization [116]. Lima and collaborators (2015) used rat models of 5-FU-induced OM to test AM or not, as a natural dressing biomaterial, during 14 or 21 days [116]. Immunostaining for PCNA and VEGF revealed increased cellular proliferation and neovascularization in the 5-FU-induced OM group treated with AM at days three and seven, the inflammatory and proliferative phases, respectively [116]. The inflammatory process was mild in the group treated with AM, where an increase in the number of CD4+ and CD8+ cells was found only in day seven. After 14 or 21 days of AM treatment, no difference was found in the number of CD4+ and CD8+ cells in the lesions of this group or in the control group without AM [116]. AM presented biocompatible properties and efficient dressing, stimulated cellular proliferation as well as promoted stable adherence onto lesions. Neovascularization was also stimulated and the wound healing process was accelerated proving that this natural scaffold might stimulate tissue regeneration and be used for clinical trials [116].

### Buccal adhesive polymer films loaded with natural products

Alginate, a natural non-toxic polysaccharide found in some brown algae species has been widely used as scaffold for drug delivery in burns and ulcerative lesions [91]. Similarly, chitosan, purified from chitin found in fungi cell walls and exoskeleton of shellfishes and insects, has been applied as biomaterial for tissue engineering [91]. Watanabe and collaborators (2013) tested the healing effect of a chitosan-alginate film containing royal jelly (RJ) in a hamster model of 5-FU-induced OM and mild abrasion of the cheek pouch [117]. RJ is a natural product secreted by hypo-pharyngeal and mandibular glands of worker honeybees (Apis mellifera) composed of proteins with anti-inflammatory and anti-oxidant effects [117]. The healing process was examined by measuring the area of mucositis and MPO activity. Films containing 10% and 30% of RJ significantly reduced ulcerations. Treatment with RJ reduced ulcerations and erythema at day 3 and 8, respectively. MPO and the TNF-α and IL-1β proinflammatory cytokines were also significantly lower in the site of injury after 8 days of treatment. The expression of KGF was not observed when films containing 10% RJ were used. *In vitro*, 10% of RJ significantly scavenged free radicals. Possibly, the polymer might contribute for the healing process due to the effective controlled release of RJ in the site of injury. The anti-inflammatory and antioxidant effects of chitosan-alginate film containing RJ reinforce its use as a promising dressing biomaterial [117].

## DISCUSSION AND FUTURE DIRECTIONS

Although tissue engineering of oral mucosa has been less developed than skin [101], Blackwood [92] was able to produce biodegradable electrospun scaffolds for dermal and oral mucosa replacement and Kumbar [118] highlighted the progress of tissue engineering of dental, hard and soft tissues such as enamel, dentin, alveolar bone, periodontium, oral mucosa and salivary glands. In this regard, for regenerative medicine purposes, the electrospinning technique produces flexible three dimensional porous membranes with a suitable structure for wound dressing and also drug delivery [119]. As these membranes regard similarities with the skin and mimic the extracellular matrix milieu, reports in the area of regenerative medicine reveal that these fibers-constituted membranes have been used for periodontal diseases due to its physical structure, which allow the addition of drugs, growth factors, and cells [118–120].

Nazarnezhad et al. (2020) reported that biodegradable electrospun membranes with the addition of vascular endothelial growth factor; angiopoetin1 (Ang1); transforming growth factor-β (TGF-β1); fibroblast growth factor (FGF) and hepatocyte growth factor (HGF); matrix metalloproteinase (MMPs); plasminogen activator inhibitor-1 (PAI-1) or nitric oxide synthase (NOS) exert effects of: stimulating angiogenesis, permeability and leukocyte adhesion; stabilizing vessels and inhibiting permeability; stimulating extracellular matrix (ECM) production; stimulating angio/arteriogenesis; matrix remodeling, release and activation of growth factors; stabilizing nascent vessels and promoting angiogenesis and vasodilation, respectively [121]. These bioactive molecules may also contribute to the healing effect of OM. In addition, these bioactive molecules could be embedded in polymers microspheres and entrapped in mucoadhesive gels to be applied as dressing materials onto OM ulcerations. For instance, Skeff et al. (2014) used a mucoadhesive gel with the addition of S-Nitrosoglutathione to accelerate recovery from 5-Fluorouracil-Induced Oral Mucositis [42]. On the other hand, Lima et al. (2015) applied only amniotic membrane, due to its natural biological components, as a biological dressing for OM ulceration healing in experimental model which stimulated tissue regeneration [116].

In the present review, we evidenced promising data for OM healing including the use of drug-loaded membranes or scaffold-loaded natural products or nanoparticles, or mucoadhesive gels loaded with microspheres as carriers of antioxidant agents. These data brings support to propose the production of novel membranes in combination with different growth factors and biomolecules, nanoparticles, natural products or drugs that favors tissue repair, following the requirements of regenerative medicine and tissue engineering applications. Novel combinations of distinct biological and chemical compounds or cells that are crucial elements to stimulate injured sites and accelerate healing and regeneration should be tested in animal models aiming to optimize OM therapies. In conclusion, we highlighted the latest boosts for OM treatments tested in experimental models and expect that these therapies be applied in the near future in clinical trials to ameliorate the side effects of chemoradiotherapies in cancer patients resulting in improvement of their quality of life.
